# *AcOTApks* Gene-Based Molecular Tools to Improve Quantitative Detection of the Mycotoxigenic Fungus *Aspergillus carbonarius*

**DOI:** 10.3390/foods14010065

**Published:** 2024-12-29

**Authors:** Angelo Agnusdei, Rita Milvia De Miccolis Angelini, Francesco Faretra, Stefania Pollastro, Donato Gerin

**Affiliations:** Department of Soil, Plant and Food Sciences, University of Bari Aldo Moro, Via Amendola 165/A, 70126 Bari, Italy; angelo.agnusdei@uniba.it (A.A.); ritamilvia.demiccolisangelini@uniba.it (R.M.D.M.A.); donato.gerin@uniba.it (D.G.)

**Keywords:** digital droplet PCR (ddPCR), quantitative PCR (qPCR), ochratoxin A (OTA), grapevine, musts

## Abstract

Ochratoxin A (OTA) is a mycotoxin, a common contaminant of grapes and their derivatives, such as wine, and classified as possible human carcinogen (group 2B) by the International Agency for Research on Cancer (IARC). *Aspergillus carbonarius* is the main producer of OTA in grapes. The stability of the molecule and the poor availability of detoxification systems makes the control of *A. carbonarius* in vineyards the main strategy used to reduce OTA contamination risk. Several molecular methods are available for *A. carbonarius* detection, but the correlation between the abundance of fungal population and OTA contamination needs to be improved. This study aimed at the development of innovative quantitative PCR (qPCR) and digital droplet PCR (ddPCR) tools to quantify the mycotoxigenic fractions of *A. carbonarius* strains on grapes, based on the key gene *AcOTApks* in the pathway of OTA biosynthesis. Different primers/probe sets were assessed, based on their specificity and sensitivity. This method allowed to quantify up to 100 fg∙µL^−1^ [cycle of quantification (Cq) = 37] and 10 fg∙µL^−1^ (0.38 copies∙µL^−1^) of genomic DNA (gDNA) from *A. carbonarius* mycelium in qPCR and ddPCR, respectively. The sensitivity as to artificially contaminated must samples was up to 100 conidia (Cq = 38) and 1 conidium (0.13 copies∙µL^−1^) with qPCR and ddPCR, respectively. Finally, the methods were validated on naturally infected must samples, and the quantification of the fungus was in both cases highly correlated (r = +0.8) with OTA concentrations in the samples. The results showed that both analytical methods can be suitable for improving the sustainable management of OTA contamination in grapes and their derivatives.

## 1. Introduction

Ochratoxin A (OTA) is a mycotoxin produced by several fungal species belonging to the genera *Aspergillus* and *Penicillium* [1], first discovered in corn meal inoculated with *Aspergillus ochraceus* [2]. Chemically characterized by a dihydroisocoumarin moiety amide-linked to L-phenylalanine [3], OTA presents high toxicity, in part due to the presence of a chlorine atom in the aromatic ring [4], and has been classified as possible human carcinogen (group 2B) by the International Agency for Research on Cancer (IARC) [5], with neurotoxic, carcinogenic, immunotoxic, genotoxic, and teratogenic effects on humans and animals [6,7,8,9]. OTA is a common contaminant of various foods, drinks and feeds [10,11,12,13,14] and was also reported in 1996 in wine [15], which is considered the second-ranking source of daily OTA intake for humans after cereals [11], and for which a limit of 2 μg∙kg^−1^ is reported [16]. OTA contamination is more frequent on grapes from warmer climates [17] and in red wines due to the maceration step during vinification process [18,19].

In grapes, the *Aspergillus* spp. of the section *Nigri* (black aspergilli) are dominant before harvest, and some of its members are known as OTA producers [20,21,22,23]. In particular, several studies report that *Aspergillus carbonarius* is the main source for OTA contamination in wine, especially because a high percentage of isolates can produce the mycotoxin [24,25], although some non OTA-producer isolates have been reported [26]. The abundance of *A. carbonarius* in the field, and consequent OTA contamination levels, can vary among different vineyards and sampling points [27,28], making risk assessment and management difficult. Furthermore, to date, few detoxification systems are available, in part because the chemical structure of OTA gives it a marked stability against heat treatments [9]. Particular attention is paid to the use of fining agents capable of adsorbing and retaining the mycotoxin on their surface; however, these systems can often alter the organoleptic quality of the wine in the face of low decontamination efficacy [4,29]. Therefore, the monitoring of OTA-contamination risk along the supply chain, starting with the first stages of production, and extending to the harvesting and winemaking phases, represents the most important strategy for ensuring wine quality and safety. Given the close correlation between the spread of *A. carbonarius* in the field and OTA contamination in the final product, an early and accurate identification and quantification of the fungus in the vineyard, as well as in other crops, is useful to prevent the risk of OTA contamination and aid in the development of new sustainable control strategies.

Different diagnostic methods for *A. carbonarius* identification were developed over the years. Firstly, a semi-selective medium based on malt extract agar added with antibiotics and the fungicide boscalid was developed; it allowed the discrimination of *A. carbonarius* from the other black Aspergilli in grape must [30]. With the increasing use of molecular diagnostic techniques, several protocols for the identification of *A. carbonarius* were reported. A classic polymerase chain reaction (PCR)-based protocol allowed the discrimination of *A. carbonarius* from *A. japonicus* using primers designed on the calmodulin gene [31]. Subsequently, the same genomic region was used for the development of a quantitative PCR (qPCR) assay using a TaqMan probe [32] and a digital droplet PCR (ddPCR) assay [33]. Compared with the other PCR-based techniques, the ddPCR allowed for some technical problems related to the low efficiency of the reaction to be overcome, in addition to difficulties related to the presence of interferers, such as tannins, polysaccharides or pigments, and as to the quantification bias related to the use of a standard curve [34,35]. In this technique, the microfluidic technology allows the partitioning of the reaction mix into thousands of water-in-oil droplets, each representing an independent nano-PCR event [36]. In this way, using binary Poisson statistics, the absolute number of target DNA copies in a sample can be calculated from the ratio of positive events (presence of PCR product) to total partitions [37,38], leading to an accurate quantification of the target, also at low copy numbers [39].

In parallel, different studies aimed at the comprehension of the OTA biosynthesis in *A. carbonarius* led to the identification and characterization of the enzymes involved in the biosynthetic pathway [40,41,42,43,44] and, in particular, the key role of a polyketide synthase which is responsible for the biosynthesis of the dihydrocumarin’s precursor has been shown by gene disruption in *A. carbonarius* [43] and similarly in *A. ochraceus* [45] and *A. westerdijkiae* [46].

The aim of this work was to develop new qPCR and ddPCR molecular diagnostic tools for the quantification of *A. carbonarius* OTA-producing strains on grape and grape derivatives, based on the *AcOTApks* (otaA) gene. Different primers/probe sets targeting the *AcOTApks* were obtained and assessed for their specificity *in silico* and on a panel of OTA-producing *Aspergillus* spp. and bacterial and fungal species commonly associated to the grape bunch. The diagnostic obtained was then assessed in terms of sensitivity and replicability, in comparation with a scorpion-based qPCR diagnostic previously developed [47] and routinely used for *A. carbonarius* detection. Furthermore, these methods were applied to quantify the abundance of *A. carbonarius* on naturally infected grape must samples collected from different vineyards in the Apulian region and showing different OTA contamination levels.

## 2. Materials and Methods

### 2.1. Isolates and Media

*Aspergillus carbonarius* AC49 (CBS 144853) was preliminarily used for the optimization of primer/probe sets in both ddPCR and qPCR. Other *A. carbonarius* and bacterial and fungal isolates commonly associated with the grape bunch, as well as OTA-producing *Aspergillus* species (Table S1) were also used to evaluate primers and probe specificity. All isolates are stored as aqueous solutions of 10% glycerol at −80 °C in the culture collection of the Department of Soil, Plant and Food Science, University of Bari Aldo Moro. Fungal isolates were routinely grown on potato dextrose agar [PDA: infusion of 200 g peeled and sliced potatoes kept at 60 °C for 1 h; 20 g of dextrose, adjusted at pH 6.5; and 20 g of agar (European bacteriological agar, LLG-labware, Meckenheim, Germany) per liter], while for the bacterial isolates, nutrient agar [NA: 13 g nutrient broth (Oxoid Ltd., Basingstoke, UK) and 20 g of agar per liter] was used. All isolates were incubated at 25 °C and in darkness.

### 2.2. DNA Extraction

The gDNA of fungal isolates was extracted from 3-day-old cultures grown on cellophane disks overlaid on PDA medium, as described in a previous work [48]. The gDNA of bacteria isolates was extracted from 2 mL overnight nutrient broth cultures (NB: NA without agar) grown at 25 °C, under shaking at 200 rpm. Briefly, the samples were centrifuged for 15 min at 4500 rpm, and the cells pellet washed with 5 mL of ultrapure sterile water. In all, 200 µL of breaking buffer [49], 400 µL of phenol:chloroform:isoamyl alcohol (25:24:1) and 50 mg of glass beads were added to the pellet before 2 min of shaking in a mixer mill (MM301 Retsch GmbH, Haan, Germany) at maximum speed. The supernatant was collected and 400 µL of phenol chloroform-isoamyl alcohol (25:24:1) was added and the DNA subsequently precipitated by addition of 20 µL of 3M sodium acetate and 500 µL of isopropyl alcohol, followed by incubation at −20 °C for 30 min. After centrifugation at 4 °C for 20 min and at 14,000 rpm, the pellet was washed with 500 µL of 70% ethanol and vacuum dried, and the DNA was resuspended in 50 µL of ultrapure sterile water.

For grape must, a modified cetyltrimethylammonium bromide (CTAB) protocol was used. Briefly, 2 mL of must were centrifuged at 14,000 rpm for 30 min, the aqueous phase removed, and 700 µL of CTAB buffer (0.12 M Na_2_HPO_4_, 1.5 M NaCl, 2% CTAB), 0.5 g of glass beads and two steel beads added to the pellet before shaking for 30 s in the mixer mill (MM301 Retsch GmbH) at maximum speed. After chloroform extraction, the clear supernatant was transferred and DNA precipitated with two volumes of isopropyl alcohol, keeping the sample at −80 °C for 30 min. The pellet obtained after centrifugation at 12,000 rpm for 15 min was washed with 200 µL of 70% ethanol, vacuum dried and resuspended in ultrapure sterile water. Finally, the DNA was purified as follows: (1) a sepharose column was obtained by puncturing a 0.5 mL micro-tube at the bottom and adding a drop of glass beads suspended in TE (10 mM Tris·Cl pH 8.0, 1 mM EDTA pH 8.0) and then 500 µL of sepharose; (2) the micro-tube was placed in a 2 mL tube and the column was obtained by conducting two consecutive centrifugations at 3000 rpm for 3 min, removing, after each centrifugation, the flow-through; and (3) the obtained sepharose column was placed into a 1.5 mL tube, and the DNA loaded and then eluted by centrifugation at 3000 rpm for 3 min.

Quality and quantity of DNA were evaluated using the Nanodrop™ 2000 spectrophotometer (Thermo Fisher Scientific Inc.) and Qubit 2.0 fluorometer (Life Technologies Ltd., Paisley, UK) with the dsDNA BR Assay kit (Thermo Fisher Scientific Inc., Wilmington, DE, USA). Samples were stored at −20 °C until use.

### 2.3. Scorpion Probe Obtainment

The *A. carbonarius* scorpion probe was drawn on sequence-characterized amplified regions (SCARs) following RAPD analysis [47] carried out on 9 isolates of *A. niger*, 5 of *A. aculeatus*, and 34 of *A. carbonarius* (Table S1). Three couples of SCARs primers which were specific for *A. carbonarius* were obtained according to the previously described protocol [50] and checked for specificity on a panel of 5 isolates of each species of *A. carbonarius*, *A. niger*, and *A. aculeatus*, as well as the others listed in Table S1. The following PCR conditions were used: 4 min at 95 °C, followed by 30 cycles of 30 s at 94 °C, 30 s at 52 °C, 45 s at 72 °C, and a final extension at 72 °C for 7 min. The reaction mix has been described before [50]. The sensitivity was assessed using gDNA extracted from the isolates of *A. carbonarius* AC12, AC16 and AC17 at 9 different concentrations (0.01, 0.05, 0.1, 0.5, 1, 5, 10, 25 and 50 ng∙µL^−^^1^), at the amplification conditions reported above. The scorpion primer was obtained from SCAR OPA3_519_C and customized by Oswel DNA service (University of Southampton, Great Britain), and then modified by the addition of fluorescent reporting dye 6-carboxyfluorescein (FAM) in 5’ position, methyl red monomer and HEG 344.1 (Table 1).

### 2.4. AcOTApks Gene-Based Primers and Probes

To obtain primers/probe sets specific for the AcOTApks, an in silico analysis was performed using the gene sequence of the isolate ITEM 5010 (v4.0; protein ID:1051847), available at https://mycocosm.jgi.doe.gov/Aspca4 (last access on 3 December 2024). The OTApks gene sequences from 22 isolates belonging to *Aspergillus* genera were aligned by SeqMan Pro (version 14.1.0, DNASTAR, Madison, WI, USA) and checked for the presence of single nucleotide polymorphisms (SNPs). Three primers/probe sets were designed using Primer3plus (https://www.bioinformatics.nl/cgi-bin/primer3plus/primer3plus.cgi (last access on 3 December 2023)), according to the droplet digital PCR manufacturing guidelines (ddPCR Application Guide, www.bio-rad.com, last access: 3 December 2024) and synthetized by Macrogen (Seoul, Republic of Korea), labelling each probe at 5′ with the fluorophore FAM and at the 3′ with the quencher BHQ1 (Table 1).

### 2.5. qPCR and ddPCR Assays

Real-time qPCR experiments were conducted in a CFX96™ Real-Time PCR Detection System Thermal Cycler (Bio-Rad Laboratories, Hercules, CA, USA). Amplification reactions with scorpion primers were conducted in a 25 µL reaction mixture containing 1× PCR Buffer without MgCl_2_ (Sigma-Aldrich, St. Louis, MO, USA), 2 mM MgCl_2_ (Sigma-Aldrich), dNTPs mix (75 µM each), 0.05 µM scorpion primer, 0.5 µM reverse primer, 1.5 U Taq DNA polymerase (Sigma-Aldrich), 1% polyvinylpyrrolidone (PVP), 1 µL of template DNA, and water up to 25 µL. Amplification conditions were 95 °C for 5 min followed by 40 cycles of 94 °C for 45 s and 55 °C for 1 min.

The *AcOTApks*-based assay amplification mixture consisted of 1× Sso Advanced™ Universal Probes Supermix (Bio-Rad Laboratories), 250 nM of each primer, 160 nM of probe, 2 μL of DNA template, and water up to 20 μL. Cycling conditions were 95 °C for 3 min, followed by 40 cycles of 95 °C for 10 s and 60 °C for 30 s. For both scorpion- and *AcOTApks*-qPCR, the Cq values, slope, efficiency (E) of the reaction, and coefficient of determination (R^2^) were calculated using the CFX Manager™ software (version 3.1, Bio-Rad Laboratories).

The ddPCR reactions were performed according to the ddPCR Application Guide (www.bio-rad.com, last access: 3 December 2024). For each sample, a water–oil emulsion was obtained by loading 20 µL of reaction mixture containing 1×ddPCR^TM^ Supermix for probes (No dUTP) (Bio-Rad Laboratories), primers and probe (500/250 nM), 1 µL of DNA sample, water up to 22 µL, and 70 µL of droplet generation oil for probes (Bio-Rad Laboratories) into an 8-channel cartridge (Bio-Rad Laboratories) covered with DG8 Gaskets (Bio-Rad Laboratories). Droplets were generated in a QX200 Droplet Generator (Bio-Rad Laboratories), and 40 µL of each sample emulsion was carefully transferred into ddPCR 96-Well PCR Plates (Bio-Rad Laboratories), sealed with a pierceable foil in a PX1™ PCR plate sealer (Bio-Rad Laboratories), and amplified in a T100™ Thermal Cycler (Bio-Rad Laboratories) with the following cycling parameters: initial denaturation at 95 °C for 10 min, followed by 40 cycles of denaturation at 94 °C for 30 s, and annealing (58–60 °C) for 1 min with a ramp rate of 2 °C∙s^−1^ and a signal stabilization at 98 °C for 10 min. The plates were finally transferred to a QX100 droplet reader (Bio-Rad Laboratories) and acquired data were analyzed by Quanta Soft™ (version 1.7.4, Bio-Rad Laboratories), manually setting up the fluorescence amplitude threshold for discrimination between positive and negative droplets. To optimize the ddPCR conditions, 58, 60, and 62 °C as annealing temperatures were preliminary assessed using the primers/probe sets 1 and 2 on samples containing 0.1, 1, and 10 ng·µL^−1^ of AC49 DNA template. All the experiments were conducted in triplicate and a no-template control (NTC, ultrapure sterile water) was always included.

### 2.6. Validation of the Assays

The specificity of the assays was assessed on 14 isolates of *A. carbonarius* collected from different vineyards, a panel of microorganisms commonly associated to wine grape bunches, fungal species phylogenetically close to *A. carbonarius*, and bacterial strains commonly used as biocontrol agents (Table S1). The analytical sensitivity of the assays was firstly evaluated by testing decimal dilutions (from 10 fg·µL^−^^1^ to 10 ng·µL^−^^1^) of *A. carbonarius* AC49 gDNA. Then, the methods were applied on grape must samples artificially contaminated with different amounts of AC49 conidia (from 10^0^ to 10^8^ conidia). The accuracy of the method was evaluated in terms of repeatability, calculating the intra-assay coefficient of variation (intra assay-CV). Finally, the validation of the methods was performed on grape must samples, of different varieties, naturally infected by *A. carbonarius* and showing different levels of OTA contamination.

### 2.7. Analysis of AcOTApks Partial Sequences and OTA Quantification

The gDNA of the *A. carbonarius* isolates AC32, AC35, AC46, AC47, AC49 and AC72 was amplified using the primer couple Seq-F1/Seq-R1 (Table 1), flanking the targeting region of set 2, and sequenced by an external service (Macrogen). The retrieved sequences were then aligned with SeqMan Pro software (version 14.1.0; DNASTAR) and checked for the presence of polymorphisms. Furthermore, OTA concentration was determined in culture broth, obtained as previously described [51], at 0, 6 and 8 days after inoculation (DAI) by HPLC analysis and normalized to mycelium dry weight. For HPLC analysis, 20 µL of culture broth, filtered on 0.22 µm syringe filters, was injected into the valve (mod. 7725I, Rheodyne, Cotati, CA, USA) of the chromatographic apparatus, which was equipped with an isocratic pump (mod. LC10AD, Shimadzu, U.S.A. manufacturing, Canby, OR, USA), a fluorometric detector (mod. RF-10AxL, Shimadzu, λex = 333 nm, λem = 460 nm) and a SCL-10Avp system controller (Shimadzu). A reversed-phase Discovery C-18 analytical column (15 cm × 4.6 mm, 5 mm particles; Supelco, Sigma-Aldrich, St. Louis, MO, USA) preceded by a SecurityGuard (Phenomenex, Torrance, CA, USA) was used for the analysis, and the extracts were identified by comparing the retention time (7.3 min) with that of the OTA standard (Supelco, Sigma-Aldrich).

### 2.8. Statistical Analysis

To compare the sensitivity of the assay, linear regression models were developed with Microsoft Excel (version 365; Microsoft Corporation, Redmond, WA, USA). Correlation analysis between *A. carbonarius* quantification and OTA amount in naturally contaminated grape must samples was performed with CoStat-software (version 4.451; CoHort Software, Monterey, CA, USA). K-means clustering analysis was conducted on OTA accumulation data, normalized to the dry weight of the mycelium by the Statistics Kingdom cluster analysis calculator (available at: https://www.statskingdom.com/cluster-analysis.html last accessed on 3 December 2024). The number of clusters was determined by the elbow method.

## 3. Results

### 3.1. Scorpion Primer Obtainment

Under the adopted conditions, 201 RAPD-PCR amplicons, separable in agarose gel into discrete bands, and corresponding to molecular sizes ranging from 100 to 1600 bp, were identified from the 48 tested isolates of *A. aculeatus*, *A. carbonarius* and *A. niger*. Most were polymorphic, and among the 12 markers specific for *A. carbonarius* the following amplicons were determined: OPA-2_800_ (800 bp originated from the primer OPA-2), OPA-3_519_ (519 bp from the primer OPA-3) and OPA-9_720_ (720 bp from the primer OPA-9); these were cloned and sequenced. Different couples of primers were designed on the sequence of each SCAR (Table 2) and assessed for their specificity against a panel of fungal species commonly associated with winegrape diseases. None of the primer pairs designed on the marker OPA-9_720_ yielded amplification products, while two couples of primers from the markers OPA-2_800_ (OPA-2_800_D) and OPA-3_519_ (OPA-3_519_C) resulted in specific determinations for *A. carbonarius*. The sensitivity of the selected primers was assessed, and the OPA 3_519_C primers pair showed the higher sensitivity, generating bands of intense fluorescence down to 5 ng·µL^−^^1^; the OPA 3_519_C (sense primer) was then transformed into the scorpion primer (Table 1).

### 3.2. AcOTApks Primers and Probe

The *in silico* study of the *OTApks* gene of 22 OTA-producing *Aspergillus* isolates allowed to identify the most promising region to utilize for designing specific primers. One couple of primers/probe (set 1) was designed on exon 5, and two couples (set 2 and set 3) were designed targeting exon 7 (Table 1), which showed the highest number of SNPs. To identify the optimal amplification conditions the primers/probe sets were firstly assessed in ddPCR using 0.1, 1, and 10 ng·µL^−1^ of *A. carbonarius* AC49 DNA as a target. Furthermore, three different annealing temperatures (58, 60 and 62 °C) were compared. Set 1 showed a total saturation of positive events that precluded the appropriate quantification of the target DNA at a concentration of 10 ng·µL^−1^. Furthermore, the primers/probe sets 2 and 3 allowed to obtain the best separation between positive and negative events, and less rainfall in the plot was achieved with an annealing temperature of 60 °C and set 3. In consequence, set 2 and set 3 were applied, with an annealing temperature of 60 °C, for the determination of the sensitivity and the specificity of the assays.

### 3.3. Evaluation of the Sensitivity

#### 3.3.1. Sensitivity of Scorpion-Based Real-Time qPCR

**Figure 1:**
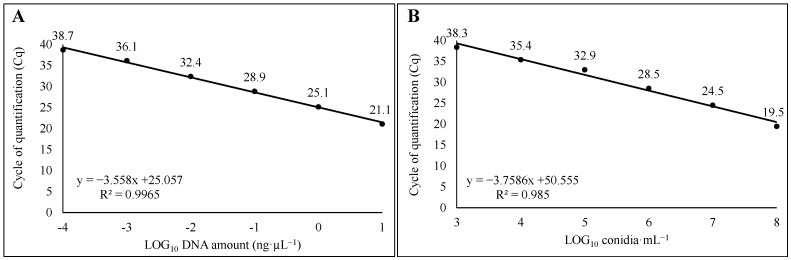
Linear regressions obtained by (**A**) Scorpion-qPCR assay on DNA from pure mycelium of *A. carbonarius* (AC49), ten-fold serial diluted from 10 fg·µL^− 1^ to 10 ng·µL^−1^, and (**B**) Scorpion-qPCR assay on DNA from musts artificially contaminated with AC49 conidial suspension, from 10^0^ to 10^8^ conidia·mL^−1^.

Ten-fold serial dilutions of DNA extracted from the mycelium of *A. carbonarius* (AC49) and grape must samples artificially contaminated with *A. carbonarius* conidia suspension were used to assess the sensitivity and linearity of the method. The limits of detection (LoD) and quantification (LoQ) were 100 fg∙µL^−1^ of target DNA at 38.7 ± 0.5 cycles and 1∙10^3^ conidia·mL^−1^ at 38.3 ± 0.0 cycles, using DNA from mycelia and from must, respectively. In both cases the assay showed a good linearity in the range of concentration assessed, with R^2^= 0.996, slope = −3.558, y intercept = 25.057 and E = 110.3%, and R^2^ = 0.985, slope = −3.758, y intercept = 50.555 and E = 81.8%, for DNA from mycelia and must, respectively (Figure 1A,B). Intra-assay CV values of 1.15% and 0.62% were retrieved when assessing DNA from mycelium and must samples, respectively.

#### 3.3.2. Sensitivity of AcOTApks-Based Real-Time qPCR

**Figure 2:**
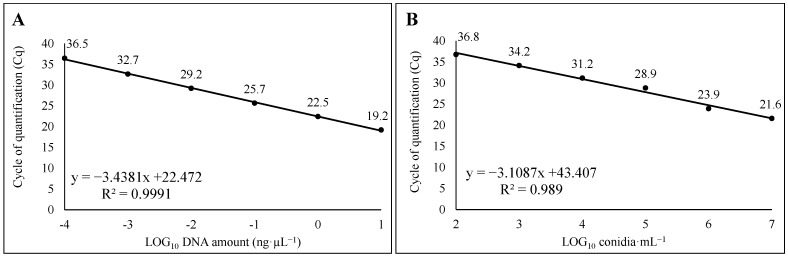
Linear regression obtained by (**A**) *AcOTApks*-based qPCR on DNA from pure mycelium of *A. carbonarius* (AC49), ten-fold serial diluted from10 fg·µL^−1^ to 10 ng·µL^−1^, and (**B**) *AcOTApks*-based qPCR assay on DNA from musts artificially contaminated, with AC49 conidial suspension from 10^0^ to 10^8^ conidia·mL^−1^.

Using primer/probe set 3, the assay allowed to identify and properly quantify (LoD and LoQ) up to 100 fg∙µL^−1^ of template DNA at 36.5 ± 0.2 cycles and 1 × 10^2^ conidia∙mL^−1^ at 36.8 ± 0.2 cycles, assessing DNA from mycelium and must, respectively. In this case, a good linearity was also observed, with R^2^ = 0.999, slope = −3.438, y intercept = 22.472 and E = 98.5%, and R^2^ = 0.989, slope = −3.109, y intercept = 43.407 and E 110.3%, using DNA from mycelia and must, respectively (Figure 2A,B). Intra-assay CV values of 0.51% and 0.38% were retrieved, respectively, for qPCR on DNA from mycelia and must. The results obtained with set 2 were similar to those for set 3 and therefore are not reported.

#### 3.3.3. Sensitivity of ddPCR Assay

**Figure 3:**
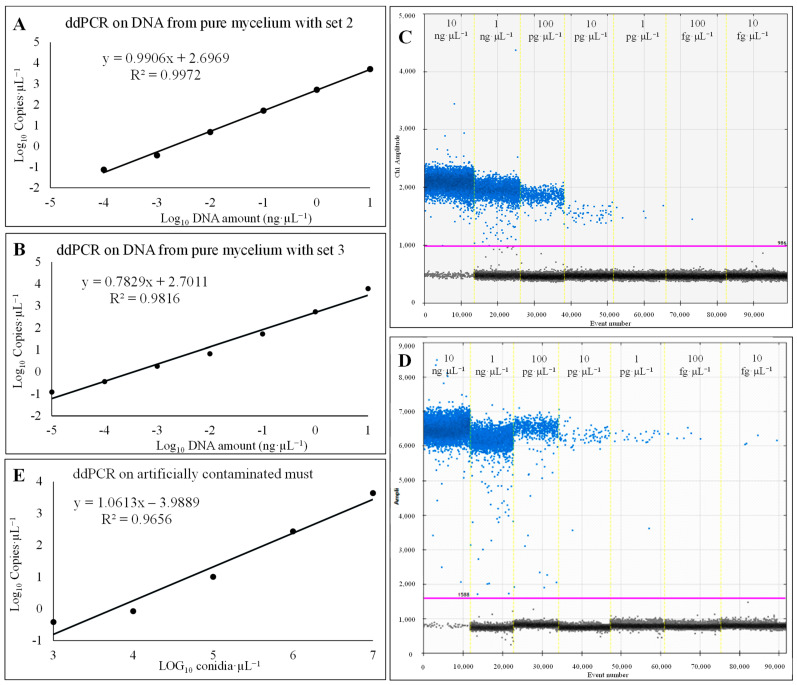
Linear regression generated assessing ten-fold serial dilution (from10 fg·µL^−1^ to 10 ng·µL^−1^) of DNA from pure mycelium of *A. carbonarius* (AC49), using (**A**) primer/probe set 2 and (**B**) primer/probe set 3. The model was elaborated by relating the logarithm of target DNA concentration to the logarithm of quantification in copies·µL^−1^. Plot obtained from ddPCR on DNA from pure mycelium of *A. carbonarius* (AC49) using (**C**) set 2 and (**D**) set 3. Blue dots represent positive events, while black dots under the threshold line (pink) represent negative events. (**E**) Linear regression model generated from ddPCR analysis of DNA from musts artificially contaminated with AC49 conidial suspension, from 10^0^ to 10^8^ conidia·mL^−1^.

The method described allowed to identify and properly quantify up to 0.07 copies∙µL^−1^, corresponding to a LoD of 100 fg∙µL^−1^ of target DNA, and 0.12 copies∙µL^−1^, corresponding to a LoD of 10 fg∙µL^−1^ of target DNA, when assessing DNA from pure mycelium using set 2 and set 3, respectively. In general, both diagnostics showed good linearity, with R^2^ = 0.997, slope = 0.9906 and intercept y = 2.6969, on the dynamic range from 100 fg∙µL^−1^ to 10 ng∙µL^−1^ for set 2 (Figure 3A), and R^2^ = 0.9816, slope = 0.7829 and intercept y = 2.7011, in a dynamic range of quantification from 10 fg∙µL^−1^ to 10 ng∙µL^−1^ for set 3 (Figure 3B). For higher concentrations, the droplets could be positively saturated, making impossible the application of a Poisson statistical algorithm, and finally resulting in a loss of linearity. The intra-CV values were 7.8% and 6.5% for set 2 and set 3, respectively. Furthermore, set 3 allowed a for better discrimination between positive and negative events, with an increase in fluorescence amplitude and a decrease in rainfall (Figure 3C,D).

When assessing DNA from contaminated must samples, set 3 allowed to quantify up to 0.13 ± 0.03 copies∙µL^−1^, corresponding to a LoD of 1∙10^0^ conidia∙mL^−1^. A good linearity was observed in the range from 1∙10^7^ to 1∙10^3^ conidia∙mL^−1^, with R^2^ = 0.965, slope = 1.061 and intercept = −3.988 (Figure 3E). For higher concentrations of target DNA, the droplets resulted positively saturated, making impossible the application of Poisson statistical analyses, while for the last two concentrations assessed, a loss of linearity was observed. An intra-assay CV of 6.8% was recorded.

### 3.4. Specificity and Evaluation of the Assays on Field Samples

A high specificity was reported for the scorpion diagnostic, with positive amplifications exclusively when *A. carbonarius* DNA was used as a target. Regarding the *AcOTApks*-based diagnostics, both primers/probe sets showed high specificity for all *A. carbonarius* isolates and only in a few cases was a slight, low aspecificity observed, corresponding to 57 copies∙µL^−1^ (10 ng∙µL^−1^ of DNA of *Cytospora vitis*) using the primer/probe set 2, and 2.9 copies∙µL^−1^ (10 ng∙µL^−1^ of DNA of *Penicillium nordicum*) with set 3. The accuracy of the method was assessed on 6 must samples collected from different fields in the Apulian region and showing different OTA contamination levels. For this aim, the correlation between *A. carbonarius* population density and OTA contamination levels was calculated. A high correlation was observed for all the assays, reaching r = 0.86 for the scorpion-qPCR and r = +0.81 for the *AcOTApks*-qPCR and -ddPCR assays, respectively (Table 3).

### 3.5. Further Investigations of the AcOTApks Polymorphisms

**Figure 4:**
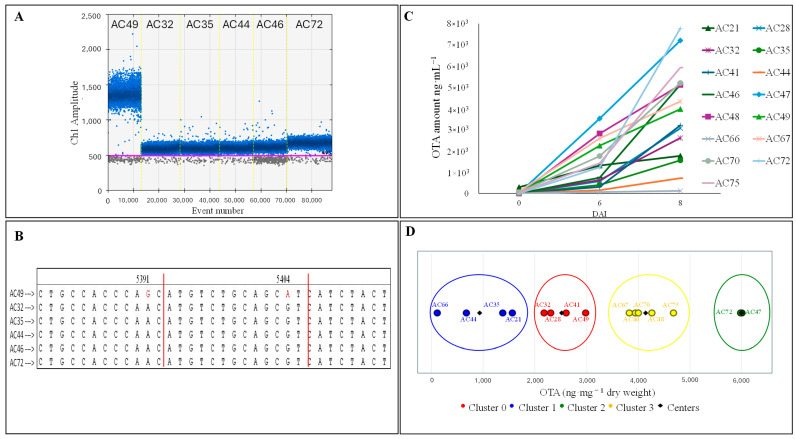
(**A**) Amplitude of fluorescence in the ddPCR plot of the droplets generated from the amplification of DNA samples from isolates AC49, AC32, AC35, AC44, AC46 and AC72. (**B**) *AcOTApks* partial genome sequences obtained with primer Seq-F1/Seq-R1. Single nucleotide polymorphisms (SNPs) in the isolates AC32, AC35, AC44, AC46 and AC72 were highlighted in positions 5931 and 5404. (**C**) Rate of ochratoxin A (OTA) production in the *A. carbonarius* isolates assessed. (**D**) Clustering of *A. carbonarius* isolates as a function of OTA accumulation at 8 days. The K-means clustering analysis categorized the 15 isolates into four groups, identified as representing 95.1% of the variability by use of the elbow method.

A lower amplitude of fluorescence with respect to *A. carbonarius* AC49 (reference strain for this study) was observed in the plot of ddPCR, when analyzing the DNA of five isolates of *A. carbonarius* (AC32, AC35, AC44, AC46 and AC72) with primers/probe set 2 (Figure 4A). This prevented the correct setting of the threshold line, hindering the discrimination between positive and negative events, and in consequence, the correct quantification of target DNA in the samples. The subsequent analysis of the partial genomic region targeted by set 2 revealed the presence of two single nucleotide polymorphisms (SNPs) in the five isolates with lower fluorescence amplitude, consisting of a substitution for a guanidine (G) with an adenine (A) and for an A with a G. After the alignment of the sequences on the *AcOTApks* gene of the isolate ITEM 5010 (jgi ID:1051847), the polymorphisms were in positions 5931 and 5404, respectively (Figure 4B), and were included in the annealing site of the probe *AcOTApks*-P2, leading to lower annealing efficiency in the first cycles of amplification and a final decrease in the amplitude of fluorescence of the droplets.

To investigate whether the presence of the polymorphisms could be related to any differences in the OTA production, OTA quantification was performed in three different time points (0, 6 and 8 DAI) using 15 isolates of *A. carbonarius* (Figure 4C). The K-means clustering analysis allowed to cluster the *A. carbonarius* isolates based on OTA accumulation, at 8 DAI, into four distinct groups, explaining 95.1% of the variance (Figure 4D). Four isolates, clustered in group 0 (AC28, AC32, AC41 and AC49), showed a range of OTA production between 2.6 and 3.9 µg·g^−^^1^. Three of them showed a higher accumulation rate between 6 and 8 DAI, while a constant trend of OTA accumulation from 0 to 8 DAI was observed in the AC49. In group 1 were clustered the four isolates (AC21, AC35, AC44 and AC66), showing the lowest OTA secretion, inclusively, between 0.1 and 1.7 µg·g^−^^1^, while two isolates (AC47 and AC72) were clustered in group 2, and showed the highest OTA production, reaching 7.7 µg·g^−^^1^. The AC47 showed a constant production of OTA during the time, while the AC72 production rate increased from 6 to 8 DAI. Finally, five isolates (AC46, AC48, AC67, AC70 and AC75) were clustered in group 3, with an OTA production range between 4.3 and 5.9 µg·g^−^^1^. Also in this case, the isolates showed different OTA accumulation trends. The five isolates showing the two polymorphisms in the genomic region of the *AcOTApks* gene studied were included in different clusters as generated by K-means analysis, and showed a different OTA accumulation trend, demonstrating there is no correlation between the presence of the polymorphisms and the production of the mycotoxin.

## 4. Discussion

OTA is one of the most dangerous mycotoxins for human health, in part due to the consideration that chronic exposure to OTA can be even more dangerous than acute exposure [5,52]. Grapes and wines represent an important source of daily OTA intake, due both to the numerous reports of contamination [53,54,55,56], especially for wine production from warm environments [57], and the increase in wine consumption at global level [58]. Since several papers have reported *A. carbonarius* as the main OTA producer on winegrapes [10,24,25,59] and the actual methods for OTA detoxification present defects to some extent [60], the rapid and accurate detection and the rational control of *A. carbonarius* in pre- and post-harvest conditions constitute the main strategy for OTA contamination risk management. Over the years, several molecular diagnostic methods have been set up for *A. carbonarius* detection, as well as for other ochratoxigenic Aspergilli [61]. In particular, a diagnostic method targeting the gene codifying for calmodulin was applied in qPCR and ddPCR [27,31]. However, since not all the isolates of *A. carbonarius* are OTA producers [26], the presence of *A. carbonarius* on grapes could be not strictly correlated with OTA contamination. Furthermore, the population dynamics of *Aspergillus* spp. could strongly vary, not only depending on the area and the vineyard and as to specific years [62], but also between different sampling points in the same vineyards [27]. For these reasons, the correlation data between the incidence on grapes of *A. carbonarius* and OTA contamination in musts could differ markedly under different conditions, especially when using primers targeting conserved regions for *A. carbonarius* quantification. Recently, the availability of the *A. carbonarius* genome allowed the identification and characterization of the genes involved in the OTA biosynthetic pathway. In particular, the deletion of a polyketide synthase resulted in the loss of OTA production [43]. Furthermore, the recent availability of the ddPCR technique allows to improve the reaction efficiency, also reducing the effects of inhibitors such as polysaccharides and tannins, with respect to which bunch samples, musts and wines are particularly rich [63,64]. Other advantages of the ddPCR include target DNA partitioning and the application of Poisson’s statistical analyses, which enable an absolute quantification of nucleic acid, overcoming the need for a standard curve [65].

In this study, a molecular diagnostic tool targeting the *AcOTApks* (otaA) gene was developed for sensitive and accurate detection of mycotoxigenic isolates of *A. carbonarius* in must and grape-derived products. Three couples of primers and probe were obtained and assessed in qPCR and ddPCR in terms of specificity, sensitivity and accuracy. The optimal ddPCR performance, in terms of best separation between positive and negative events and less rainfall in the plot, was achieved with a primer/probe concentration of 500/250 nM and an annealing temperature of 60 °C. The primers/probe set 3 (only in few cases) showed a slight low aspecificity (2.9 copies∙µL^−1^ assessing 10 ng∙µL^−1^ of DNA of *Penicillium nordicum*), which nonetheless did not influence the quantification of *A. carbonarius*, because the amount of DNA used (10 ng∙µL^−1^) corresponded to infection levels that cannot happen in the field. Set 3 allowed to properly quantify up to 10 fg∙µL^−1^, corresponding to 0.38 copies∙µL^−1^ of target DNA from *A. carbonarius* mycelium, and up to 1 conidium of *A. carbonarius*, corresponding to 0.13 copies∙µL^−1^, in artificially contaminated grape must samples, resulting in determinations 10 times and 100 times more sensitive than *AcOTApks*-based qPCR that allowed to quantify up to 100 fg∙µL^−1^ of *A. carbonarius* gDNA at 36.5 cycles and 1∙10^2^ *A. carbonarius* conidia∙mL^−1^ at 36.8 ± 0.2 cycles. These values are in line with those commonly observed in the development of ddPCR and qPCR assays for the quantification of different plant pathogens, including *Aspergillus* spp. [66,67,68,69,70], and comparable to other innovative techniques such as Loop-Mediated Isothermal Amplification (LAMP) [71].

The partitioning of the target DNA into individual micro-reactions makes ddPCR much more sensitive than qPCR and reduces susceptibility to inhibitors, ensuring a distinguishable positive signal; this is also the case when moderate PCR inhibition occurs in a droplet [72].

A good linearity was retrieved in a dynamic range of quantification from 10 fg∙µL^−1^ to 10 ng∙µL^−1^ and from 10^3^ up to 10^7^ conidia∙mL^−1^, assessing DNA from mycelium (R^2^ = 0.9816) and artificially contaminated must (R^2^ = 0.965), respectively. For higher concentration of target DNA, the droplets resulted positively saturated, making impossible the application of Poisson-based statistical analyses. The upper quantification limit represents a key limit of the ddPCR, as also reported by other authors [38,73]. A preliminary evaluation of the method was conducted on naturally infected must samples, showing different OTA contamination levels, in comparation with a scorpion-based qPCR assay previously developed and routinely used for *A. carbonarius* detection and quantification [47]; the data relating to the development of the latter are reported in this paper. The abundance of *A. carbonarius*, as determined with the different techniques, was high (r > +0.8) and significantly (*p* ≤ 0.05) correlated with OTA contamination in the same samples, demonstrating that this assay can be successfully used to quantify the OTA-producing population of *A. carbonarius*.

The analysis of OTA production at three time points (0, 6 and 8 DAI) allowed to highlight differences in OTA production among 15 *A. carbonarius* isolates collected from different vineyards in the Apulia region. The K-means clustering analysis allowed to categorize the isolates into four groups showing different OTA accumulations at 8 DAI. The different behavior of *A. carbonarius* isolates with respect to the production of OTA, as well as the presence of conserved SNPs in the *AcOTApks* gene, more common in the OTA-producing strains compared with strains not producing OTA, are known [26,51], and this may explain the lack of complete correlation between *A. carbonarius* abundance and the OTA contamination data. In this study, five isolates showing two SNPs in a coding region of the *AcOTApks* were identified; however, the K-means clustering analysis confirmed that the presence of the polymorphisms does not determine differences in OTA production. Since the *AcOTApks* primers and probes were designed on a coding region (exon 7) of the *AcOTApks*, this assay could represent a reference for further studies, in addition to its potential application with respect to cDNA samples in one-step reverse transcription–Droplet Digital PCR [74]. This could lead to an accurate quantification of the mycotoxigenic fraction of *A. carbonarius,* improving the relation of *A. carbonarius* population dynamic on grapes with the OTA amounts in musts and wine samples.

## 5. Conclusions

Molecular diagnostics for use in qPCR and ddPCR, targeting the key gene *AcOTApks* (otaA) of the OTA biosynthetic pathway, were developed to quantify the mycotoxigenic fraction of *A. carbonarius* strains on wine grapes and grape derivatives. The *AcOTApks* qPCR and ddPCR were both specific and showed high sensitivity and good reliability. The abundance of *A. carbonarius* determined with these techniques in naturally contaminated must samples was highly correlated with OTA contamination in the same samples, proving that these diagnostics can successfully be applied in the quantification of the mycotoxigenic fraction of *A. carbonarius* population on grapes. Furthermore, since the *AcOTApks* primers and probes target a coding region on exon seven of the *AcOTApks*, this assay could represent a reference for further application on cDNA samples, improving the relation of the *A. carbonarius* population dynamic on grapes with the OTA amounts in musts and wine samples.

## Figures and Tables

**Table 1:** Features of primers and probes used in the work.

Set No.	Name	Sequence (5′-3′)	Amplicon Size (bp)
1	*AcOTApks-F1*	CCACGAGTACGACCGAGTCAA	101
	*AcOTApks-R1*	CACTTGCCATGGCCGATT	
	*AcOTApks-P1*	FAM-TGACCGCATTCCAC-BHQ1	
2	*AcOTApks-F2*	ACGAGGGCGTCAACGAGAT	101
	*AcOTApks-R2*	CCCATAACGGGACGAGTAGATG	
	*AcOTApks-P2*	FAM-ACCCAGCATGTCTGCA- BHQ1	
3	*AcOTApks-F3*	GACCAGGAGTTGCGGAAT	60
	*AcOTApks-R3*	CTCGTCGGTGTCGTCAA	
	*AcOTApks-P3*	FAM-CGGTCTTCAATCCCGGCTTCTT- BHQ1	
**-**	Seq-F1	ACCTCATGTGTTCCCCTCTG	570
	Seq-R1	GCAGAATAAAATTGACGCCGTC	
**-**	Scorpion Probe	FAM-GCCGCATTCCCTGCATTGCATTGCAATTGAGGCGGC**76**GTGTATCCTGCTCTGAATCC-3′	157
	OPA-3_519_C-Rev	CTATCAACCTCGACTACTTCC	

7: MR (methyl red monomer); 6: HEG (hexethylene glycol).

**Table 2:** Features of primers designed on sequence-characterized amplified regions (SCARs).

RAPD Marker	Primer Couple	Primer Sequence		Expected Product
		Forward (5′→3′)	Reverse (5′→3′)	Length (bp)
OPA-2_800_	A	TTTCTCCTATCTACTCCGTACC	CACCTATCAATGTCTGACACC	112
	B	TCCTATCTACTCCGTACC	CTATCAATGTCTGACACC	105
	C	CTTTTTCTCCTATCTACTCC	CTATCAATGTCTGACACC	112
	D	GTGTTTTTCTCCTATCTACTCC	ATACTCAAGCTATGCATCC	478
OPA-3_519_	A	GCAGAGATCCTTAGATCC	AAGCTACGAGTAAACATCC	145
	B	TGTATCCTGCTCTGATCC	TCAACCTCGACTACTTCC	153
	C	GTGTATCCTGCTCTGATCC	CTATCAACCTCGACTACTTCC	157
	D	CACAGCAGAGATCCTTAGATCC	AGACTCTCATCAATTATCGACG	308
OPA-9_720_	A	TGAGTAAGAGTATCGTGGTGGGGG	TTGGGGTGTTCAATCCAGGGTC	150
	B	TGACCCTGGATTGAACACC	GACCATGATTACGCCAAGC	173
	C	ACACCCCAACATTATTAGG	TTTCACACAGGAAACAGC	180
	D	TGACCCTGGATTGAACACC	GACCATGATTACGCCAAGC	293
	E	GGAATTCGATTGTGTGCC	AAATGTAGGCCCCAACTC	119
	F	GAGTTGGGGCCTACATTT	CCAGGGTCATGAAACACC	299

**Table 3:** Correlation coefficient (r) between ochratoxin A contamination and *A. carbonarius* population density in the musts.

Assay	Correlation Coefficient (r)
Scorpion qPCR	+0.86 *
*AcOTApks* qPCR	+0.81 *
*AcOTApks* ddPCR	+0.81 *

* r values significant for *p* ≤ 0.05.

## Data Availability

The original contributions presented in this study are included in the article/supplementary material. Further inquiries can be directed to the corresponding author(s).

## Supplementary Materials

The following supporting information can be downloaded at: https://www.mdpi.com/article/10.3390/foods14010065/s1, Table S1: Strains used in this work.
