# Supplementary material for: AcOTApks Gene-Based Molecular Tools to Improve Quantitative Detection of the Mycotoxigenic Fungus Aspergillus carbonarius
Source: Foods. 2024 Dec 29;14(1):65. doi: 10.3390/foods14010065 (PMC11719998; doi:10.3390/foods14010065)
Supplement: Supplementary file 1 [file foods-14-00065-s001.zip › foods-3381078-supplementary.pdf]

Table S1 Strains used in this work

Species (strain)	Experiment	Host	Locality	Deposition year	Protein ID*
Alternaria alternata (Aa1)	c	Cherry	Turi (Ba, Italy)		-
Aspergillus aculeatus (Aa 100)	a	Vine grapes-Negramaro	Sanpancrazio (Br, Italy)	2000	-
Aspergillus aculeatus (Aa 101)	a	Vine grapes-Primitivo	Torricella (Ta, Italy)	2000	-
Aspergillus aculeatus (Aa 96)	a, c	Vine grapes-Primitivo	Sava (Ta, Italy)	2000	-
Aspergillus aculeatus (Aa 98)	a	Vine grapes-Primitivo	Manduria (Ta, Italy)	2000	-
Aspergillus aculeatus (Aa 99)	a	Vine grapes-Uva della scala	Locorotondo (Ba, Italy)	2000	-
Aspergillus affinis (CBS 129190)	b	-	-	-	573234
Aspergillus albertensis (IBT 14317)	b	-	-	-	89370
Aspergillus alliaceus (ITEM 4548)	c	Agro-Food microbial Culture collection ITEM- ISPA-CNR **	-	2001	-
Aspergillus alliaceus (CBS 536.65)	b	-	-	-	320960
Aspergillus awamori (ITEM 7098)	c	Agro-Food microbial Culture collection ITEM- ISPA-CNR **	-	2004	-
Aspergillus carbonarius (AC21)	a, c	Vine grapes-Primitivo	Sava (Ta, Italy)	2000	-
Aspergillus carbonarius (AC28)	a, c	Vine grapes-Negramaro	Leverano (Le, Italy)	2000	-
Aspergillus carbonarius (AC31)	a	Vine grapes-Primitivo	Sava (Ta, Italy)	2000	-
Aspergillus carbonarius (AC32)	a, c	Vine grapes	Manduria (Ta, Italy)	2000	-
Aspergillus carbonarius (AC33)	a	Vine grapes-Primitivo	Torricella (Ta, Italy)	2000	-
Aspergillus carbonarius (AC34)	a	Vine grapes-Aleatico	Leverano (Le, Italy)	2000	-
Aspergillus carbonarius (AC35)	a, c	Vine grapes-Negroamaro	Sandonaci (Br, Italy)	2000	-
Aspergillus carbonarius (AC36)	a	Vine grapes-Syrah	Leverano (Le, Italy)	2000	-
Aspergillus carbonarius (AC37)	a	Vine grapes-Fiano	Locorotondo (Ba, Italy)	2000	-
Aspergillus carbonarius (AC38)	a	Vine grapes-Primitivo,	Sava (Ta, Italy)	2000	-
Aspergillus carbonarius (AC39)	a	Vine grapes-Malvasia	Locorotondo (Ba, Italy)	2000	-
Aspergillus carbonarius (AC40)	a	Vine grapes	-	2000	-

Aspergillus carbonarius (AC41)	a, c	Vine grapes-Italia	Castellaneta (Ta, Italy)	2000	-
Aspergillus carbonarius (AC42)	a	Vine grapes	Locorotondo (Ba, Italy)	2000	-
Aspergillus carbonarius (AC43)	a	Vine grapes-Aglianico	Locorotondo (Ba, Italy)	2000	-
Aspergillus carbonarius (AC44)	a, c	Vine grapes-Susumaniello	Locorotondo (Ba, Italy)	2000	-
Aspergillus carbonarius (AC45)	a	Vine grapes-Susumaniello	Locorotondo (Ba, Italy)	2000	-
Aspergillus carbonarius (AC46)	a, c	Vine grapes-Gaglioppo	Locorotondo (Ba, Italy)	2000	-
Aspergillus carbonarius (AC47)	a, c	Vine grapes	Locorotondo (Ba, Italy)	2000	-
Aspergillus carbonarius (AC48)	a, c	Vinegrapes-Primitivo	Torricella (Ta, Italy)	2000	-
Aspergillus carbonarius (AC49)	a, b, c	Vine grapes-Negroamaro	Leverano (Le, Italy)	2000	-
Aspergillus carbonarius (AC51)	a, c	Vine grapes-Negramaro;	Sanpancrazio (Br, Italy)	2000	-
Aspergillus carbonarius (AC53)	a	Vine grapes-Negramaro	Leverano (Le, Italy)	2000	-
Aspergillus carbonarius (AC55)	a	Vine grapes-Verdeca	Locorotondo (Ba, Italy)	2000	-
Aspergillus carbonarius (AC56)	a	Vine grapes-Greco Bianco	Locorotondo (Ba, Italy)	2000	-
Aspergillus carbonarius (AC57)	a	Vine grapes-Notardomenico	Locorotondo (Ba, Italy)	2000	-
Aspergillus carbonarius (AC58)	a	Vine grapes	Locorotondo (Ba, Italy)	2000	-
Aspergillus carbonarius (AC59)	a	Vine grapes	Locorotondo (Ba, Italy)	2000	-
Aspergillus carbonarius (AC60)	a	Vine grapes	-	2000	-
Aspergillus carbonarius (AC64)	a	Vine grapes-Galioppo	Cirò (Cr, Italy)	2000	-
Aspergillus carbonarius (AC65)	a	Vine grapes-Negroamaro	Leverano (Le, Italy)	2000	-
Aspergillus carbonarius (AC66)	a	Vine grapes-Primitivo	Leverano (Le, Italy)	2000	-
Aspergillus carbonarius (AC67)	a, c	Vine grapes-Sangiovese	Leverano (Le, Italy)	2000	-
Aspergillus carbonarius (AC68)	a	Vine grapes-Montepulciano	Leverano (Le, Italy)	2000	-
Aspergillus carbonarius (AC70)	c	Vine grapes-Aglianico	Locorotondo (Ba, Italy)	2000	-
Aspergillus carbonarius (AC72)	c	Vine grapes-Montepulciano	Locorotondo (Ba, Italy)	2000	-
Aspergillus carbonarius (AC75)	c	Vine grapes-Primitivo	Torricella (Ta, Italy)	2000	-

Aspergillus carbonarius (AC82)	a	Vine grapes-Negramaro	-	2000	-
Aspergillus carbonarius (AC83)	a	Vine grapes-Negramaro	-	2000	-
Aspergillus carbonarius (AC84)	a	Vine grapes-Primitivo	Sava (Ta, Italy)	2000	-
Aspergillus carbonarius (AC87)	a	Vine grapes-Primitivo	Torricella (Ta, Italy)	2000	-
Aspergillus carbonarius (ITEM 5010)	b	-	-	-	1051847
Aspergillus cretensis (CBS 112802)	b	-	-	-	260161
Aspergillus elegans (CBS 116.39)	b	-	-	-	404109
Aspergillus flavus (ITEM 4591)	c	Agro-Food microbial Culture collection ITEM- ISPA-CNR **	-	2001	-
Aspergillus flocculosus (CBS 112785)	b	-	-	-	239064
Aspergillus muricatus (CBS 112808)	b	-	-	-	167277
Aspergillus nakazawaec (CBS 640.78)	b	-	-	-	230931
Aspergillus neoauricomusc (CBS112787)	b	-	-	-	171425
Aspergillus niger (An1)	a, c	Vine grapes-Primitivo	Sava (Ta, Italy)	-	-
Aspergillus niger (An2)	a, c	Vine grapes-Primitivo	Torricella (Ta, Italy)	2000	-
Aspergillus niger (An3)	a, c	Vine grapes-Aglianico	Acerenza (Pt, Italy)	2000	-
Aspergillus niger (An4)	a, c	Vine grapes-Aglianico	Acerenza (Pt, Italy)	2000	-
Aspergillus niger (An5)	a, c	Vine grapes-Aglianico	Acerenza (Pt, Italy)	2000	-
Aspergillus niger (An6)	a	Vine grapes-Aglianico	Acerenza (Pt, Italy)	2000	-
Aspergillus niger (An8)	a	Vine grapes-Uva di Troia	Andria (BAT, Italy)	2000	-
Aspergillus niger (An9)	a	Vine grapes-Bombino nero	Andria (BAT, Italy)	2000	-
Aspergillus niger (An11)	a	Vine grapes-Negramaro	Sava (Ta, Italy)	2000	-
Aspergillus niger (ATCC 13496)	b	-	-	-	294293
Aspergillus niger (CBS 513.88)	b	-	-	-	167298
Aspergillus niger (lacticoffeatus) (CBS 101883)	b	-	-	-	403593

Aspergillus ochraceus *(ITEM 4211)*	c	Agro-Food microbial Culture collection ITEM- ISPA-CNR **	-	2000	-
Aspergillus pulvericola (CBS 137327)	b	-	-	-	487357
Aspergillus roseoglobulosus (CBS112800)	b	-	-	-	176392
Aspergillus sclerotiicarbonarius (CBS 121057)	b	-	-	-	436295
Aspergillus sclerotioniger *(ITEM 7560)*	c	Agro-Food microbial Culture collection ITEM- ISPA-CNR **	-	2006	-
Aspergillus sclerotioniger (CBS 115572)	b	-	-	-	574133
Aspergillus sesamicola (CBS 137324)	b	-	-	-	339267
Aspergillus steynii *(ITEM 17280)*	c	Agro-Food microbial Culture collection ITEM- ISPA-CNR **	-	2016	-
Aspergillus steynii (IBT 23096)	b	-	-	-	380337
Aspergillus subramanianii (CBS 138230)	b	-	-	-	300036
Aspergillus tubingensis *(ITEM 4496)*	c	Agro-Food microbial Culture collection ITEM- ISPA-CNR **	-	2001	-
Aspergillus welwitschiae *(ITEM 6126)*	c	Agro-Food microbial Culture collection ITEM- ISPA-CNR **	-	2002	-
Aspergillus welwitschiae (CBS 139.54b)	b	-	-	-	180635
Aspergillus westerdijkiae *(ITEM 18000)*	c	Agro-Food microbial Culture collection ITEM- ISPA-CNR **	-	2018	-
Aspergillus westerdijkiae (CBS 112803)	b	-	-	-	296166
Bacillus amyloliquefaciens (FZB24)	c	Commercial (TAEGRO)	-	-	-
Bacillus subtilis (qst 713)	c	Commercial (Serenade Max)	-	-	-
Botrytis cinerea (SAS 56)	c	Monosporic	-	-	-
Cladosporium sp.	c	Pomegranate	Rutigliano (Ba, Italy)	2018	-
Colletotrichum gloeosporioides	c	Tangerine	Castellaneta (TA, Italy)	2017	-
Cytospora vitis	c	Vine grapes-1103 Paulsen	Otranto (Le, Italy)	2001	-
Penicillium digitatum	c	-	-	-	-
Penicillium expansum	c	-	-	-	-
Penicillium nordicum	c	-	-	-	-

Penicillium paneum	c	-	-	-	-
Phomopsis viticola (PV1)	c	Vine grape-Regina	Ortanova (Fg, Italy)	1994	-
Rhizopus sp.	c	Abelia	Monopoli (Ba, Italy)	2022	-

*Accession number for the *OTApks* protein in the DOE Joint Genome Institute-Mycocosm database.

** The isolates were provided by the Agro-Food microbial Culture collection of the ISPA-CNR

Letters indicate the application: a) SCAR identification; b) *AcOTApks* primers and probes obtainment; c) Specificity assay.
